# Non-Invasive Prenatal Test Analysis Opens a Pandora’s Box: Identification of Very Rare Cases of SRY-Positive Healthy Females, Segregating for Three Generations Thanks to Preferential Inactivation of the XqYp Translocated Chromosome

**DOI:** 10.3390/genes15010103

**Published:** 2024-01-16

**Authors:** Cristina Politi, Katia Grillone, Donatella Nocera, Emma Colao, Michelle Li Bellisario, Sara Loddo, Giorgia Catino, Antonio Novelli, Nicola Perrotti, Iuliano Rodolfo, Paola Malatesta

**Affiliations:** 1 Medical Genetics, Renato Dulbecco University Hospital, Viale T. Campanella 115, 88100 Catanzaro, Italy; politicristina89@gmail.com (C.P.); k.grillone@unicz.it (K.G.); donatellanocera.dn@gmail.com (D.N.); e.colao@materdominiaou.it (E.C.); michelle.bellisario@alice.it (M.L.B.); perrotti@unicz.it (N.P.); p.malatesta@materdominiaou.it (P.M.); 2 Laboratory of Medical Genetics, Translational Cytogenomics Research Unit, Bambino Gesù Children Hospital, Scientific Institute for Research, Hospitalization and Healthcare (IRCCS), 00146 Rome, Italy; sara.loddo@opbg.net (S.L.); giorgia.catino@opbg.net (G.C.); antonio.novelli@opbg.net (A.N.); 3 Department of Human Health, University Magna Graecia of Catanzaro, Campus S. Venuta, Viale Europa, Località Germaneto, 88100 Catanzaro, Italy

**Keywords:** t(X;Y) unbalanced translocation, SRY-positive females, preferential X-inactivation

## Abstract

The translocation of the testis-determining factor, the SRY gene, from the Y to the X chromosome is a rare event that causes abnormalities in gonadal development. In all cases of males and females carrying this translocation, disorder of sex development is reported. In our study, we described a peculiar pedigree with the first evidence of four healthy females from three generations who are carriers of the newly identified t(X;Y)(q28;p11.2)(SRY+) translocation with no evidence of ambiguous genitalia or other SRY-dependent alterations. Our study was a consequence of a Non-Invasive Prenatal Test (NIPT) showing a sexual chromosomal abnormality (XXY) followed by a chorionic villus analysis suggesting a normal karyotype 46,XX and t(X;Y) translocation detected by FISH. Here, we (i) demonstrated the inheritance of the translocation in the maternal lineage via karyotyping and FISH analysis; (ii) characterised the structural rearrangement via chromosomal microarray; and (iii) demonstrated, via Click-iT^®^ EdU Imaging assay, that there was an absolute preferential inactivation of the der(X) chromosome responsible for the lack of SRY expression. Overall, our study provides valuable genetic and molecular information that may lead personal and medical decisions.

## 1. Introduction

Translocations between the X and Y chromosomes may arise during paternal meiosis [1] and, based on the breakpoint and the size of translocated regions, this crossover is able to cause different phenotypic consequences [2]. X/Y translocations usually involve the short arms (p) of the chromosomes (Xp and Yp) causing the translocation on X of the SRY (Sex-Determining Region Y) gene [2], which encodes for the testis-determining factor (TDF). However, since this is a rare event, only few cases of SRY translocation on the X chromosome are reported in the literature [3,4,5] and in all patients, abnormalities in gonadal development and infertility have been described. Males with 46, XX (SRY-positive) karyotypes present disorder of sex development (DSD) [6,7], gonadal tumours [8], testicular disease with deficiency in growth hormone [4], abnormal levels of sexual hormone and then infertility [5]. In these patients, masculinisation, despite the 46,XX karyotype, is led by the presence of SRY [1]. The incidence of 46,XX with DSD has been estimated to occur in around 1/20.000 newborn males, among which the unbalanced X/Y translocations frequently involve the Xp23.3 region [9]. Rare cases of females with 47,XXY (SRY-positive) karyotypes are reported and are associated with testicular feminization [3] and ambiguous genitalia [10].

**Figure 1:**
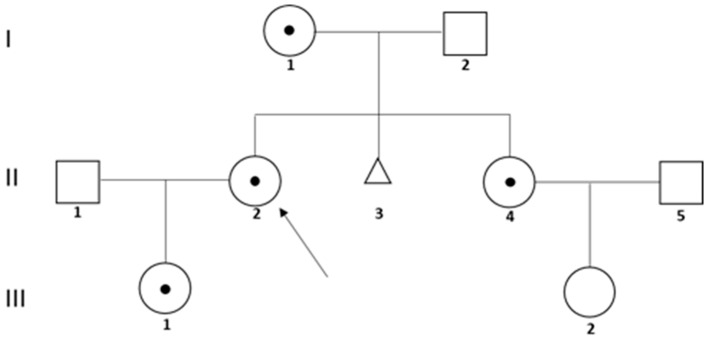
Pedigree of the family. Arrow indicates the first patient enrolled in our study (proband (II,2) that carried the translocation under investigation. The females I,1, II,4 and III,1 are carriers of the same translocation.

Here we investigated a newly identified t(X;Y)(q28;p11.2)(SRY+), detected for the first time on four healthy females belonging to three generations of the same family (Figure 1). The proband (II,2) required genetic counselling to clarify the risk of recurrence of a rare translocation found in her daughter (III,1) via Non-Invasive Prenatal Test (NIPT) analysis followed by karyotyping and FISH performed on the chorionic villi. The lack of the expected sexual abnormalities due to the presence of the SRY gene in III,1 rekindled our interest in exploring the translocation more deeply. Progressively, we investigated the proband and her mother and sister (I,1 and II,4) via conventional karyotype and FISH to analyse the segregation of the translocation and then we performed molecular studies to explain the lack of the expected masculinization.

## 2. Materials and Methods

### 2.1. Samples

After genetic counselling, an informed consent form regarding the use of biological material and results for diagnostic and research purposes was signed by each patient involved in this study. The whole peripheral blood samples from the adult women were processed at the Medical Genetics Unit of Mater Domini Hospital of Catanzaro (Catanzaro, Italy) and Chromosomal Microarray Analysis (CMA) was performed at Children’s Hospital “Bambino Gesù” (Rome, Italy). Whole peripheral blood samples were collected in heparin vacutainers (Becton Dickinson, NJ, USA) for cytogenetic analysis and in EDTA vacutainers (Becton Dickinson, NJ, USA) for molecular studies. Genomic DNA was extracted using Nuclear Laser Medicine S.r.l kit (Milano, Italy).

### 2.2. Conventional Karyotyping

Chromosome analysis was performed on at least 20 metaphases from peripheral blood lymphocytes and results were described in accordance with the ISCN (International System for Human Cytogenomic Nomenclature) 2020 [11]. Karyotypes were obtained from PHA-stimulated lymphocytes according to standard protocols. Conventional G-banding and analysis (>500-band resolution) was performed as previously reported [12]. 

### 2.3. Fluorescence In Situ Hybridization (FISH) to Characterize Chromosomal Rearrangement

FISH was conducted on fixed metaphases according to the manufacturer’s protocols by using a DXZ1 probe (spectrum green; Vysis (Abbott Laboratories, Chicago, IL, USA)) to identify the centromere region of the X chromosome and an LSI-SRY probe (spectrum orange; Vysis (Abbott Laboratories, Chicago, IL, USA)) to identify the SRY sequence (chrY:2779319-2901305) (Yp11.2; GRCh38/hg38).

### 2.4. Sanger Sequencing

Sanger sequencing of the SRY gene and its putative promoter sequence was performed. The detailed sequences of the primers used for the amplification are indicated in the Supplementary Material (Tables S1 and S2). PCRs amplifying the promoter region of SRY were performed as described by Miyamoto et al. [13]. Purified amplicons from all PCR reactions were subjected to Sanger sequencing via SeqStudio Genetic Analyser (Thermo Fisher Scientific, Waltham, MA, USA) following a standard protocol.

### 2.5. Chromosomal Microarray Analysis (CMA)

CMA was performed on DNA extracted from peripheral blood of patient II,2 using an Infinium CytoSNP-850K BeadChip (Illumina, San Diego, CA, USA), following the manufacturer’s instructions. Array scanning data were generated by the NextSeq 550 system (Illumina, San Diego, CA, USA)) and results were analysed by Bluefuse Multi software v. 4.4. All results were reported according to the GRCh38/hg38 assembly.

### 2.6. Detection of X-Inactivation Pattern and FISH Analysis

The protocol reported by Sisdelli et al. [14] was applied to identify the skewed X inactivation pattern, using the 5-ethynyl-2′-deoxyuridine (EdU) as the labelled nucleoside analogue of thymidine that was incorporated during DNA synthesis to enable the detection of late replication regions. After pre-treatment of cultured lymphocytes with EdU, FISH analysis was then conducted via LPT XYPR/G green probe (Cyto Cell, OGT, Cambridge, UK), specific for both Xp and Yp. EdU incorporation was detected by Click-iT^®^ EdU Imaging Kits (Invitrogen, Thermo Fischer Scientific, Waltham, MA, USA) according to the manufacturer’s protocol using Alexa-Fluor 467 dye to catalyse the cropper reaction between the alkyne group of EdU and the azide group of the selected fluorochrome. Inactivated X chromosomes (Xi) appeared in the microscopical examination with a stronger pink coloration, while Xp or Yp regions appeared as green signals on both arms of der(X). Fluorescent images were captured by a DMRA fluorescent microscope (Leica Microsystems, Wetzlar, Germany) with a magnification of 100×.

## 3. Results

### 3.1. Identification of a Rare t(X;Y)(q28;p11.2)(SRY+) Translocation in Healthy Females from Three Generations of the Same Family

The first patient enrolled in our study was II,2 in Figure 1, who required genetic counselling to know the risk of recurrence of a rare translocation found in her daughter (III,1), via Non-Invasive Prenatal Test (NIPT) and chorionic villus analyses.

NIPT is a Next-Generation Sequencing (NGS)-based method enabling the identification of the most common genetic abnormalities in cell-free foetal DNA (cffDNA) circulating in maternal blood [15]. This prenatal test, performed on case II,2 concerning her daughter (III,1), unexpectedly detected a high risk of sexual chromosomal anomaly, suggestive for the presence of two X chromosomes and a partial Y chromosome (XXY). The subsequent chorionic villus sampling (CVS) on III,1 showed a normal female karyotype (46,XX). However, FISH analysis on metaphases identified an SRY-positive translocation t(X;Y)(q28;p11.2). The patient II,2 decided, after appropriate and nondirective counselling, to continue pregnancy. The newborn child III,1 was healthy and without sexual ambiguity or other phenotypic alterations potentially related to the presence of SRY on the long arm (q) of chromosome X. 

In order to discriminate the rise of a de novo alteration from the presence of an inherited translocation on III,1, we performed karyotyping analysis on both non-consanguineous healthy parents, II,1 and II,2.

**Figure 2:**
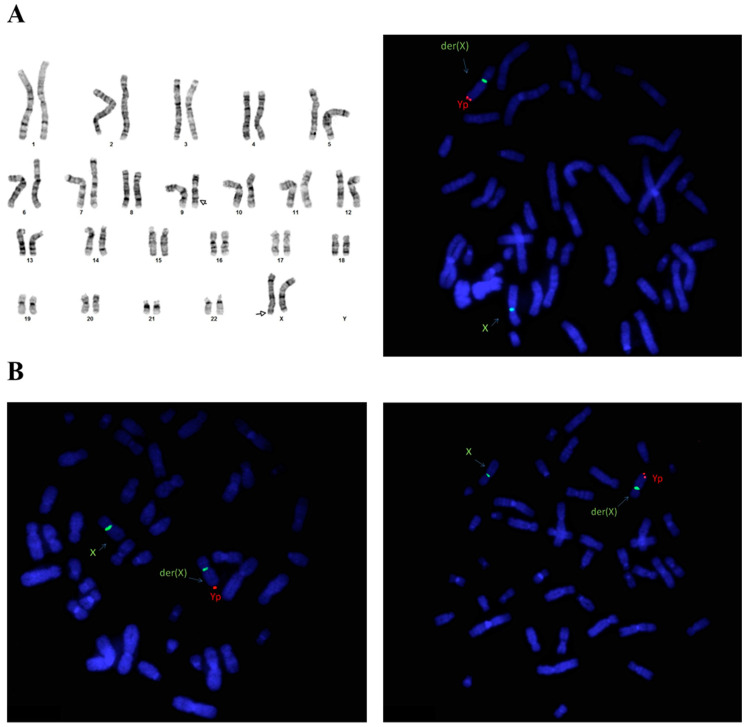
(**A**) Karyotype test of II,2 (550 band resolution, magnification 100×) showing a sexual chromosomal anomaly reported as 46,X,der(X)?add(X)(q28), a pericentric inversion of chromosome 9 as chromosome heteromorphism was also detected (left); FISH analyses conducted on II,2 (right). Red signal refers to SRY region (spectrum orange LSI-SRY probe) while green signal refers to the centromere region of the X chromosome (spectrum green DXZ1 probe); (**B**) FISH analysis conducted on I,1 and II,4 (left and right, respectively) with the same probes mentioned for II,2. FISH analysis identified a derivative of an SRY-positive X chromosome arising from a translocation t(X;Y)(q28;p11.2) on all the investigated females.

The karyotype of II,1 was normal (46,XY), whereas II,2 showed a 46,XX karyotype with a structural abnormality in one of the two X chromosomes, in correspondence with the Xq28 band (Figure 2A, left). Via FISH analysis, we found that the mother (II,2) was carrying t(X;Y)(q28;p11.2)(SRY+) (Figure 2A, right), and thus karyotyping and FISH tests were extended on the maternal lineage. Supplementary Figure S1A,B report the karyograms of I,1 and II,4, in which we observed the presence of an abnormal Xq. FISH analysis confirmed the same chromosomal rearrangement in both the mother and sister of II,2 (Figure 2B, left and right, respectively).

**Figure 3:**
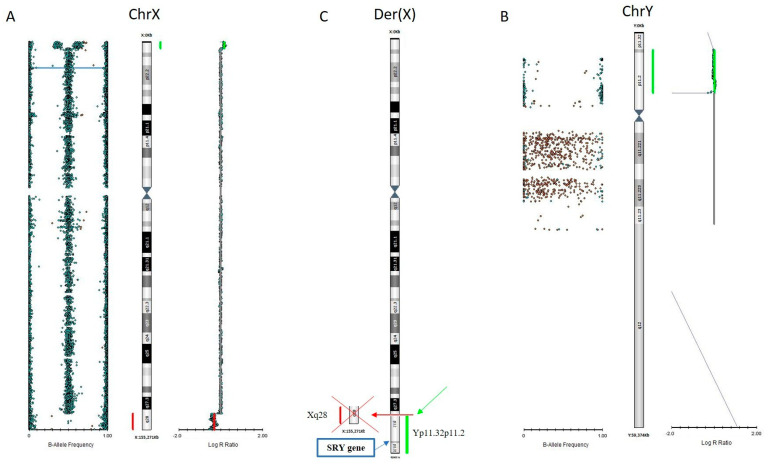
CMA profile showing allele frequency and copy number change for DNA extracted from peripheral blood sample of II,2. (**A**) The red bar indicates the deleted region at terminal q-arm of the X chromosome (Xq28) and green bar represents duplicated probes mapping in shared pseudoautosomal region (PAR1) between Xp and Yp. The allelic profile based on the B allele frequency confirmed the copy number changes. (**B**) Green bar shows additional genomic material arising from Yp spanning from pseudoautosomal region (Yp11.32) to Y-specific region (Yp11.2) for about 9 Mb. (**C**) Ideogram demonstrates that the derivative X chromosome consists of Yp11.32p11.2 replacing the Xq28 deleted portion and includes the SRY gene, mapping in Yp11.2 (blue square).

Chromosomal microarray analysis (CMA) was then performed on the genomic DNA of the proband (II,2) to characterize the translocation t(X;Y) by mapping the breakpoint. The CMA test revealed a Xq28 deletion of about 6.3 Mb and the presence of additional genomic material from the Yp11.32p11.2 region, extended about 9 Mb, that was likely translocated to the terminal q-arm of the derivative X chromosome replacing the Xq28 deleted portion (Figure 3).

CMA results are reported according to ISCN 2020 as follows: arr[GRCh38] Xq28(149765051_156007082)x1,Yp11.32p11.2 (10814_9260790)x1.

Xq28 deletion (X:149765051-156007082 bp; GRCh38/hg38) involved 188 RefSeq genes of whom 35 OMIM Morbid, whereas the Yp additional region (Y:10814-9260790 bp) encompassed 108 RefSeq genes, 4 OMIM Morbid, including SRY (Yp11.2; OMIM *480000).

We then interrogated publicly available human genomic databases (such as DECIPHER, gnomAD and ClinGen) in order to predict the potential effect induced by the loss of the genes included in the deleted Xq28 region. According to the haploinsufficiency (HI) and probability of loss-of-function intolerance (pLI) scores, we identified a list of eight genes that are essential for cell fitness (Table S3).

### 3.2. Investigation of Molecular Mechanisms Underlying the Lack of Phenotype Associated with the Presence of SRY on the X Chromosome of Healthy Females

Since all women included in the study exhibited a normal female phenotype, we investigated the molecular mechanisms underlying the lack of effect on sexual differentiation, despite the presence of SRY. We first confirmed, using Sanger sequencing (performed on genomic DNA from the proband (II,2)), a wild-type sequence in the SRY putative promoter sequence, as described by Miyamoto et al. [13], and in the correspondence of 5′UTR, 3′UTR, exonic sequence and intronic–exonic junctions.

**Figure 4:**
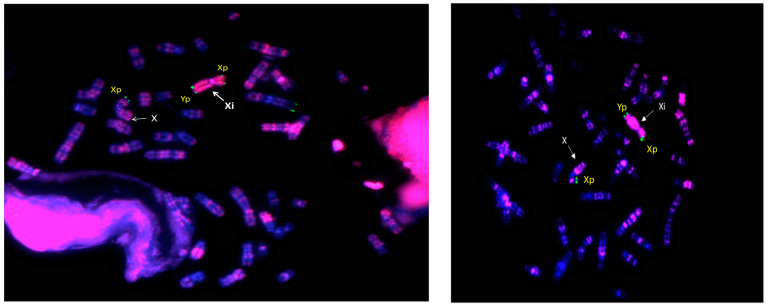
der(X) inactivation test conducted by FISH analysis on fixed metaphases pre-treated with EdU fluorescent solution on II,2 (**left**) and II,4 (**right**) patients. Green signals indicate Xp and Yp subtelomeric regions and pink signal generated by conjugating with Alexa Fluor 647 identifies der(X) as inactive (strong pink coloration) in all analysed metaphases (>25 for each patient). In all FISH analyses, chromosomes were counterstained with DAPI (blue) and images were acquired at a magnification of 100× with a DMRA fluorescent microscope.

We then investigated the X-inactivation pattern of II,2 and II,4 using the method described by Sisdelli et al. [14], followed by FISH analysis (Figure 4, left and right, respectively). Notably, in all informative metaphases analysed for both patients (>25 per sample), we observed a preferential inactivation of the SRY+ derivative X chromosome (Figure 4).

## 4. Discussion

Our study is focused on the genetic and molecular characterization of a newly identified t(X;Y)(q28;p11.2)(SRY+) translocation found in four healthy females belonging to three generations of the same family. We deeply analysed three out four carriers of this rare translocation (I,1; II,2 and II,4 in Figure 1), and demonstrated its inheritance through karyotyping and FISH analyses. All the analysed women presented normal height and no physical or intellectual disabilities. The anamnestic information provided during genetic counselling highlighted a miscarriage in case I,1, and difficulties in conceiving after the first pregnancy and early menopause in II,4. However, all carriers of the Xq/Yp translocation showed a normal sexual differentiation. The translocation of the SRY gene on the X chromosome usually involves the short arm of X [2], and to the best of our knowledge, only one case of Xq28/Yp11.2 translocation has been reported to date, and is linked to a Y-positive XX true hermaphrodite with ovotesticular DSD [16]. Therefore, our cases, carrying a very uncommon Yp translocation on the long arm of X, are the first evidence of 46,XX SRY+ females without sexual ambiguity. This evidence prompted us to investigate the molecular basis for the lack of the expected masculinisation. X/Y translocations may be associated with substantial phenotypic diversity [17]; in particular, the variability in the sexual phenotype in 46,XX (SRY+) patients could be explained by different levels of X inactivation or by a position effect [2]. In our study, once the presence of the entire wild-type promoter and coding sequence of SRY were established by Sanger sequencing, we demonstrated, by FISH conducted on EdU pre-treated lymphocytes, a preferential inactivation of the X chromosome carrying the translocation on 100% of metaphases analysed for two carriers (II,2 and II,4). Since the deleted Xq region (assessed by CMA) includes haploinsufficient genes, according to HI DECIPHER and pLI scores, we hypothesize protective mechanisms that counteract the detrimental effect of Xq deletion by transcriptional hyperactivation of those genes in the active X chromosome, as reported in the literature [18]. On the other hand, this evidence of haploinsufficiency suggests that the transmission of a derivative X chromosome to men is incompatible with life, thus conferring a higher probability of having daughters [50% of probability (of which 25% with a 46,X,der(X) genotype and 25% with 46,XX) vs. 25% of probability of a 46, XY]. This speculation is in agreement with the observation that there are no cases of male sons in the maternal lineage under analysis. Moreover, the 6.3 Mb deletion of the Xq28 region includes genes whose loss has been related to premature ovarian failure (POF) [19,20,21,22,23]. Therefore, the genetic and molecular information identified by our study, when accompanied by adequate and informative genetic counselling, could have an important impact on the perspective view of preventive medicine. Indeed, knowing the link between the Xq28 region deletion and POF, the II,2 patient may schedule gynaecological check-ups to monitor her daughter’s ovarian development and, from a future perspective, they can evaluate the preservation of ovarian eggs. Regarding the II,4 patient, she failed to conceive for a second time and failed a Medically Assisted Procreation (MAP) procedure. Unfortunately, she was subjected to our analysis when she was in menopause. If she had known about the study in advance, she could have requested preimplantation genetic testing during MAP, at least to prevent potential miscarriages due to the presence of a 46Y,der(X) karyotype. Clearly, given the implications, the presence of the translocation should also be investigated in her daughter. 

Our results point out the opportunity to integrate high-throughput tests like NIPT with gold-standard procedures like FISH to obtain a comprehensive characterization of patients’ genetic landscape. As a matter of fact, on one hand, the NGS-based methods provide highly sensitive and specific information about panels of well-known and also novel genetic variants that also need to be validated, and on the other hand, FISH analysis gives the opportunity to observe the presence of selected genes directly on chromosomes [24], helping the formulation of a clinical hypothesis by providing unambiguous confirmatory results. Thus, our study underlines the importance of expanding the application of NGS-based analyses in routine practice as complementary approaches to improve the investigative scenario for diagnostic purposes. 

## 5. Conclusions

We reported, for the first time, different cases of SRY-positive females whose health and normal sexual development seem to be explained by the preferential inactivation of the Xq/Yp translocated chromosome. It is important to note that the occurrence of a preferential over a random inactivation is a rare event, likely related to the large deletion on the X chromosome region involved in the translocation. The identification of the rare t(X;Y)(q28;p11.2) translocation as an “incidental finding” of the NIPT investigation focuses attention on the crucial role of pre-test counselling to prepare the patient for a serendipitous discovery and to correctly interpret results by explaining the limits and power of each technique, and above all, on the role of post-test counselling to provide valuable information that may lead to personal and medical decisions.

## Data Availability

The data presented in this study are available on request from the corresponding author (Rodolfo Iuliano, iuliano@unicz.it).

## Supplementary Materials

The following supporting information can be downloaded at: https://www.mdpi.com/article/10.3390/genes15010103/s1, Figure S1: Karyograms of I,1 and II,4 subjects; Table S1: Primers used to amplify the SRY gene; Table S2: Primers used to amplify SRY gene promoter; Table S3: List of haploinsufficient genes.
