# Supplementary material for: Non-Invasive Prenatal Test Analysis Opens a Pandora’s Box: Identification of Very Rare Cases of SRY-Positive Healthy Females, Segregating for Three Generations Thanks to Preferential Inactivation of the XqYp Translocated Chromosome
Source: Genes (Basel). 2024 Jan 16;15(1):103. doi: 10.3390/genes15010103 (PMC10815901; doi:10.3390/genes15010103)
Supplement: Supplementary file 1 [file genes-15-00103-s001.zip › genes-2794149-supplementary.pdf]

genes

Case Report

Supplementary Material for:

Non-Invasive Prenatal Test Analysis Opens a Pandora's Box: Identification of Very Rare Cases of SRY-Positive Healthy Females, Segregating for Three Generations Thanks to Preferential Inactivation of the XqYp Translocated Chromosome

Cristina Politi [1,†], Katia Grillone [1,†], Donatella Nocera [1], Emma Colao [1], Michelle Li Bellisario [1], Sara Loddo [2], Giorgia Catino [2], Antonio Novelli [2], Nicola Perrotti [1,3], Iuliano Rodolfo [1,3,*] and Paola Malatesta [1]

[1] Medical Genetics, Renato Dulbecco University Hospital, Viale T. Campanella 115, 88100 Catanzaro, Italy; politicristina89@gmail.com (C.P.); k.grillone@unicz.it (K.G.); donatellanocera.dn@gmail.com (D.N.); e.colao@materdominiaou.it (E.C.); michelle.bellisario@alice.it (M.L.B.); perrotti@unicz.it (N.P.); p.malatesta@materdominiaou.it (P.M.)

[2] Laboratory of Medical Genetics, Translational Cytogenomics Research Unit, Bambino Gesù Children Hospital, Scientific Institute for Research, Hospitalization and Healthcare (IRCCS), 00146 Rome, Italy; sara.loddo@opbg.net (S.L.); giorgia.catino@opbg.net (G.C.); antonio.novelli@opbg.net (A.N.)

[3] Department of Human Health, University Magna Graecia of Catanzaro, Campus S. Venuta, Viale Europa, Località Germaneto, 88100 Catanzaro, Italy

* Correspondence: iuliano@unicz.it.

† These authors contributed equally to this work.

Supplementary Tables

Table S1: primers used to amplify the SRY gene

Primer name	Sequence	Amplicon Size
Primer 1 (Fw)	5’-GGGGTACCTCAACACCCCCTCAAC-3’	244 bp
Primer 2 (Rev)	5’-CGGGATCCAATTCATATAGCTTTTTGTGTCC-3’	

Table S2: primers used to amplify SRY gene promoter

Primer name	Sequence	Amplicon size
Primer 1 (Forward -Fw)	5’- TTTCGAACTCTGGCACCTTT -3’	373 bp
Primer 2 (Reverse-Rev)	5’-GCCATTTTTCGGCTTCAGTA-3’	
Primer 3 (Fw)	5’-ATCAGCAAGCAGCTGGGATA-3’	372 bp
Primer 4 (Rev)	5’-TGTAGCCAATGTTACCCGATT-3’	

Table S3: haploinsufficient genes. HI score between 0-10 (range 1-100) and pLI score ⩾ 0.9 (range 0-1) are indicative of haploinsuffiency

Gene	Description	Function	% HI	pLI
FLNA	Filamin A	Linking of actin filaments to membrane glycoproteins	8.00	1.00
MECP2	methyl-CpG binding protein 2	Regulation of gene expression	1.38	0.89
L1CAM	L1 cell adhesion molecule	Regulation of neural cell adhesion	15.58	1.00
NSDHL	NAD(P) dependent steroid dehydrogenase-like	Involved in colesterol synthesis	64.88	0.96
MTM1	Myotubularin 1	Lipid phosphatase	12.54	1.00
SLC6A8	Solute carrier family 6 member 8	Creatine:sodium symporter	22.63	0.99
ABCD1	ATP binding cassette subfamily D member 1	Transporter of very long chain fatty acid (VLCFA)-CoA	59.98	0.98
DKC1	Dyskerin pseudouridine synthase 1	Catalitic subunit of rRNA	6.32	1.00

Supplementary Figure

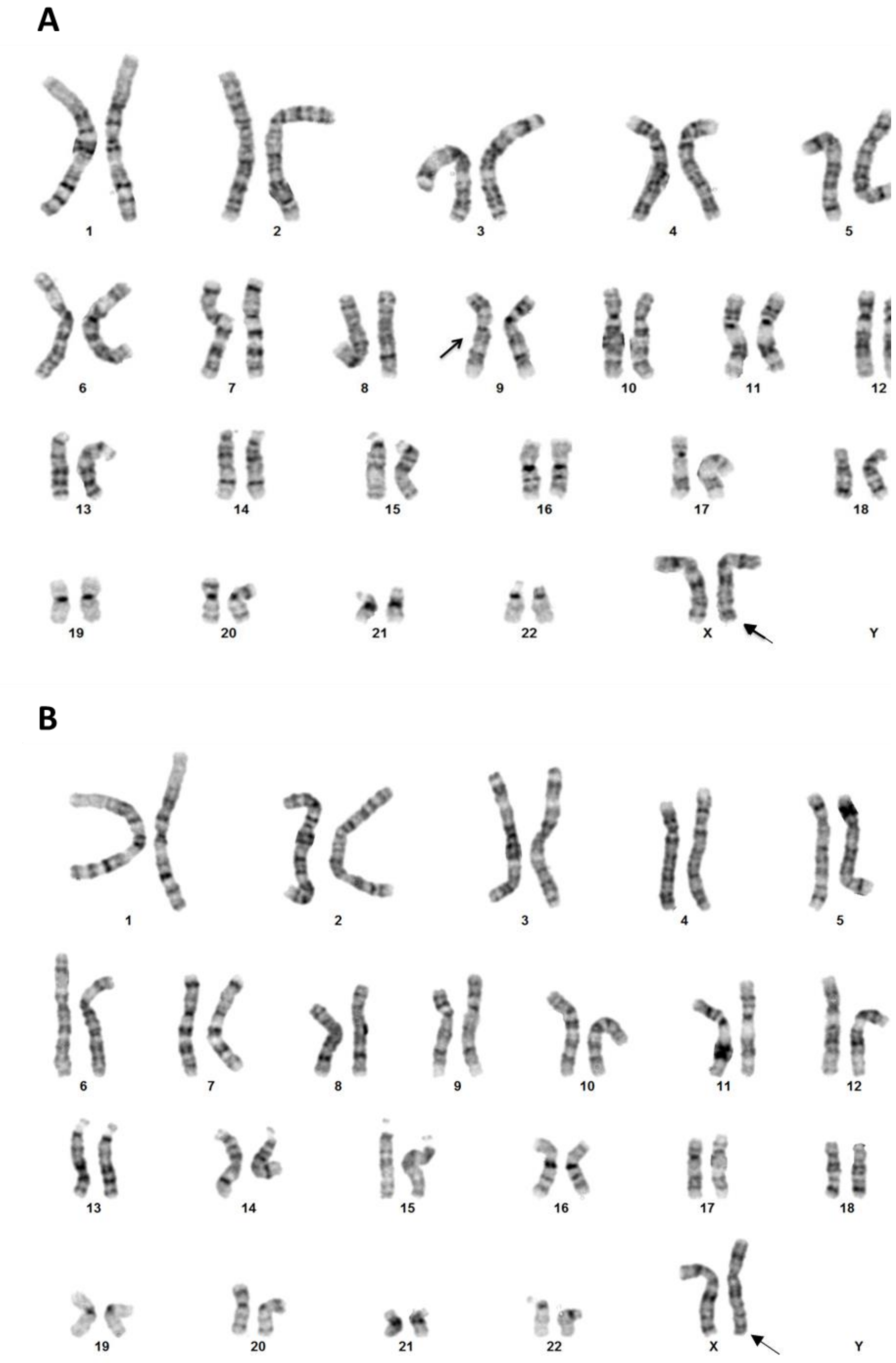

Figure S1: Karyotyping analyses (Magnification 100X, >500 band resolution). (**A**) karyogram of I,1: 46,X,der(X)?add(X)(q28), a pericentric inversion of chromosome 9 as chromosome heteromorphism was also detected (**B**) karyogram of II, 2: 46,X,der(X)?add(X)(q28).mat
